# Long-term effects of COVID-19 in diabetic and non-diabetic patients

**DOI:** 10.3389/fpubh.2022.963834

**Published:** 2022-08-15

**Authors:** Ricardo Wesley Alberca, Yasmim Álefe Leuzzi Ramos, Nátalli Zanete Pereira, Danielle Rosa Beserra, Anna Cláudia Calvielli Castelo Branco, Raquel Leão Orfali, Valeria Aoki, Alberto Jose da Silva Duarte, Maria Notomi Sato

**Affiliations:** Laboratorio de Dermatologia e Imunodeficiencias (LIM-56), Departamento de Dermatologia, Faculdade de Medicina FMUSP, Institute de Medicina Tropical da Universidade de Sáo Paulo, Sáo Paulo, Brazil

**Keywords:** SARS-CoV-2, COVID-19, complications, glycemic control, infection

## Abstract

The literature presents several reports of the impact of glycemic control and diabetes in the inflammatory and coagulatory response during coronavirus disease 2019 (COVID-19). Nevertheless, the long-term impact of the COVID-19 in diabetic patients is still to be explored. Therefore, we recruited 128 patients and performed a longitudinal analysis on COVID-19-associated biomarkers of patients with COVID-19, tree and 6 months after COVID-19 recovery and put into perspective the possible long-term complication generated after COVID-19. In our investigation, we failed to verify any long-term modification on inflammatory biomarkers, but detected an increase in the glycemia and glycated hemoglobin in patients without any pre-existing history or diagnosis of diabetes (non-diabetic patients). Although diabetic and non-diabetic patients presented elevated levels of glycated hemoglobin, the c-peptide test indicated a normal beta cell function in all patients.

## Introduction

The severe respiratory coronavirus-2 (SARS-CoV-2) infection can lead to a potentially deadly disease named coronavirus disease 2019 (COVID-19). COVID-19 death rate is higher in elderlies (1) and individuals with comorbidities (2, 3).

COVID-19 can generate a systemic inflammatory and coagulation disorder, which is exacerbated in patients with a previous history of comorbidities such as Diabetes Mellitus (DM) (4).

Several systemic biomarkers are used to assess patients' progression and organs' damage, such as creatinine, urea, c-reactive protein, neutrophil-to-lymphocyte ratio, and platelet count in the blood (5). Similar to other infections is expected that those biomarkers return to regular levels after the SARS-CoV-2 clearance, but a recent report identified an increase in inflammatory cytokines and abnormal immune function up to 8 months after COVID-19 (6).

DMinduces a low-grade systemic inflammation, increasing cytokine and reactive oxygen species production, which paradoxically curbs the anti-viral immune response (7). In addition glycemic control can influence the COVID-19 progression, with patients with more stable blood glucose levels presenting lower mortality rates (8).

Non-DM patients with SARS-CoV-2 infection can also present elevated blood glucose levels, which is associated with increased severity and mortality risk (8). Several drugs used for inflammation control can induce hyperglycemia (8), nevertheless blood glucose should return to normal levelsafter the withdrawn of the medication.

Overfifty long-term effects of SARS-CoV-2 infection have been identified until now, with the most prevalent being fatigue, headache, attention disorder, hair loss, and dyspnea (9). In addition, a manuscript identified that recovered COVID-19 patients were newly diagnosed with diabetes (10).

A recent manuscript identified the onset of diabetes pos-COVID-19 in 14 studies, in recovered patients across all age groups (11). Therefore, in this perspective manuscript we investigate and discuss the potential long-term effects of COVID-19 on inflammatory biomarkers and glycemic control on diabetic and non-diabetic patients.

## Materials and methods

This is a cohort observation study in patients from the special ward for COVID-19 patients in the Hospital das Clínicasfrom the Faculty of Medicine from the University of São Paulo (HCFMUSP). Inclusion criteria: SARS-CoV-2 RNA detection by reverse-transcriptase polymerase chain reaction in a nasal swab. The DM group consisted of patients with diagnoses of type 2 DM prior to COVID-19. The Non-DM group consisted of patients without DM diagnose previous to SARS-CoV-2 infection. Prior diagnosis of diabetes was defined as a prior diagnosis of diabetes type 2 prior to COVID-19 by any physician, whether in our hospital or another hospital. Exclusion criteria: the presence of neoplasia, immunodeficiencies or other co-infections. Four hundred forty-eight patients with positive diagnosis for COVID-19 were investigated, all patients from the cohort were instructed to return for further evaluation, but only 128 fulfilled all the inclusion criteria. All 128 patients returned for the 3- and 6-months follow-up, 72 non-diabetic patients (Non-DM) and 56 DM patients. Data in this investigation were collected from medical records. Laboratory analysis was performed at the Central Laboratory of Hospital das Clinicas, Faculdade de Medicina da Universidade de São Paulo (Divisão de Laboratório Central—HC FMUSP), and included: complete blood counts (CBC), coagulogram, liver enzymes (alanine aminotransferase—ALT and aspartate aminotransferase—AST), C-reactive protein, activated partial thromboplastin time, platelets, gamma glutamyl transferase, urea, creatinine, SARS-CoV-2 IgG, glucose, glycated hemoglobin and c-peptide.

This investigation was approved by the Ethics Committee of HCFMUSP (no.30800520.7.0000.0068-2020) and performed in conformity with the 2013 revision of the Declaration of Helsinki. Data from the hospital admission (HA), the hospital discharge (HD), and from the return for evaluation 90 (±10days) and 180 days (±10days) post-hospital discharge are presented. Normality test identified the samples as non-parametric, therefore all dataare shown as median and 95% confidence interval (C.I.). Statistical analyses were performed using the Mann-Whitney test with GraphPad Prism 9 software (GraphPad Inc., USA). *P*-value of <0.05 was considered to be statistically significant.

## Results

In our cohort, 72 non-DM patients (38 males and 34 females) with median age 62 years old (interquartile range 14) and 56 diabetic patients (34 males and 22 females) with median age 60.5 years old (interquartile range 15) (*p* = 0.7541) were hospitalized with COVID-19, on the first COVID-19 wave in Brazil, and returned for evaluation 90 and 180 days post-hospital discharge.

**Figure 1:**
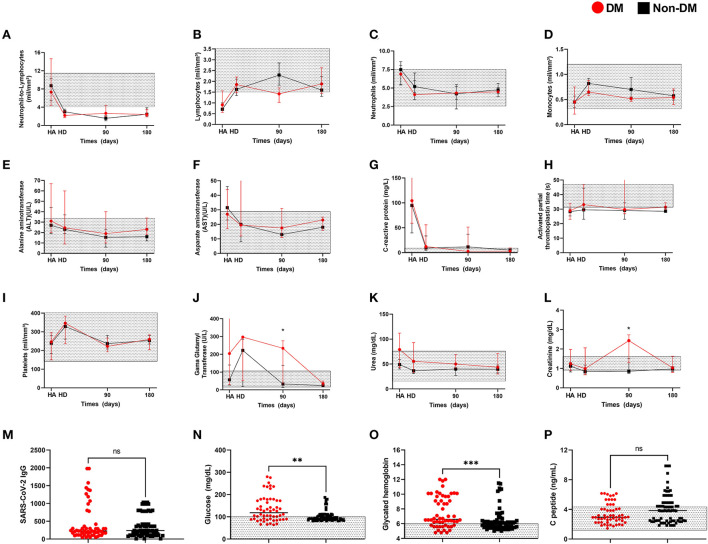
Laboratory data from Diabetic and non-diabetic COVID-19 from hospital admission, hospital discharge and up to 240 after hospital discharge. Patients' **(A)** neutrophil-to-lymphocyte ratio, **(B)** lymphocytes, **(C)** neutrophils, **(D)** monocytes, **(E)** alanine aminotransferase, **(F)** aspartate aminotransferase, **(G)** C-reactive protein, **(H)** activated partial thromboplastin time, **(I)** platelets, **(J)** gama glutamyl transferase, **(K)** urea, **(L)** creatinine from first hospitalization day, hospital discharge and monthly evaluations after SARS-CoV-2 clearance and hospital discharge. **(M)** anti-SARS-CoV-2 IgG, **(N)** fasting blood glucose, **(O)** glycated hemoglobin, **(P)** peptide C test from patients over 180 days after hospital discharge. * *p* < 0.05. Non-parametric Mann-Whitney test was used for comparisons.

No difference was observed in inflammatory hallmarks of SARS-CoV-2 infection in hospital admission (HA), hospital discharge (HD) and post-hospitalization (90 and 180 days post-HD) such as neutrophil-to-lymphocyte ratio, lymphocyte, neutrophil, monocyte count, aspartate aminotransferase (AST), alanine aminotransferase (ALT), and c-reactive protein between DM and Non-DM patients (Figures 1A–G). Similarly, coagulation biomarkers such as activated partial thromboplastin time and platelets counts also presented similar values on HA, HD as post-HD in DM and Non-DM patients (Figures 1H,I). Notably, DM and Non-DM patients presented neutrophil-to-lymphocyte ratio, lymphocyte, neutrophil, monocyte count, AST, ALT, c-reactive protein, activated partial thromboplastin time and platelets returned to within reference ranges post-COVID-19 (Figures 1A–I).

Gama glutamyl transferase levels were similar between DM and Non-DM patients in HA, HD, and 180 days time point (Figure 1J), but DM patients values were increased in comparison to Non-DM patients in the 90 days time point (Figure 1J). Urea was similar between groups in all time points (Figure 1K) and creatinine levels were onlysignificantly increased in DM patients in comparison to Non-DM patients in the 90 days time point (Figures 1L,M).

Next, we aimed to determine the long-term effects of COVID-19, we focused only on alteration 180 days after SARS-CoV-2 clearance and hospital release. Both groups presented similar levels of anti-SARS-CoV-2-specific IgG (Figure 1M). As expected, we verified thatthe DM group presented an increase in fasting blood glucose (median = 118.5; 25% percentile = 88.5; 75% percentile = 172.8) in comparison to Non-DM patients (median = 94; 25% percentile = 87; 75% percentile = 105) (*p* = 0.0019) (Figure 1N). Nevertheless, over 35% (25 patients) of Non-DM patients presented over the reference levels for fasting blood glucose (Figure 1N). Of those 25 patients with elevated blood glucose levels, only five were hospitalized in the ICU.

Next, we verified an increase in glycated hemoglobin in the DM group (median 6.6; 25% percentile = 5.8; 75% percentile = 9.7) in comparison to the Non-DM group (median = 6; 25% percentile = 5.5; 75% percentile = 6.4) (*p* = 0.0003), nevertheless 50% (36 patients) of the Non-DM group presented above the reference values for glycated hemoglobin (Figure 1O). Of those 36 patients, only five were hospitalized in the ICU (same patients with elevated blood glucose levels).

In addition, c-peptide levels were similar between the DM group (median 2.94; 25% percentile = 2.29; 75% percentile = 4.06) and the Non-DM group (median = 3.84; 25% percentile = 2.46; 75% percentile = 4.9) (*p* = 0.14), and within or above the reference values in both groups (Figure 1P).

## Discussion

In our investigation, we verified no long-term inflammatory alterations on DM and non-DM patients post-COVID-19. Nevertheless, patients from both groups presented alterations in glucose metabolism, but normal levels of C-peptide, a widely used methods for assessing pancreatic beta cell function.

Patients with Diabetes Mellitus are considered at high risk of developing a severe form of COVID-19 (4). Several investigations have postulated that the low-grade inflammation generated by high blood glucose impairs the immune response and cellular metabolism (4, 7), especially in patients with other associated comorbidities such as obesity and hypertension (12). COVID-19 death rate is higher in individuals over 65 years old (1), but our cohort mean age was below the 65 year old range in both investigated groups.

In our cohort, we identified no difference in the evaluated biomarkers at the HA and HD. In the 90 day post-hospitalization we identified an increase ingammaglutamyl transferase in the DM group in comparison to the Non-DM group, but other liver enzymes such as ALT and AST were similar between groups. Previous investigations have identified an alteration in gamma glutamyl transferase as a marker for metabolic syndrome and DM (13), which could corroborate to the elevation in this biomarker.

Creatinine levels were also increased in the DM group in comparison to the Non-DM group in the 90 days post-hospitalization. Previous reports have identified an increase in acute kidney injury during COVID-19 and recommended the long-term follow-up on kidney-associated biomarkers to identify possible sequelae (14).

Nevertheless, all patients presented similar levels in the 180 post-hospitalization time points and values within the reference range, indicating that COVID-19 does not induce chronic low-level inflammation.

In our investigation, over 6 months after SARS-CoV-2 infection all patients presenteda moderate titer of SARS-CoV-2-specific IgG. Theseresults corroborate with the similar inflammatory biomarkers during COVID-19, since several reports have identified differences in antibody titers post-COVID-19 in moderate, severe, and asymptomatic patients (15).

Long-COVID is a new syndrome characterized by the manifestations of functional, metabolic, coagulatory, or inflammatory dysfunctions post-COVID-19 (6). The mechanism behind the respiratory sequelae in long-COVID-19 can be explained by the lung injury due to the exacerbated immune response to the SARS-CoV-2. In addition, the infection by SARS-CoV-2 several chemokines and pro-inflammatory cytokines induce systemic cell activation and organs damage (16).

The SARS-CoV-2 infection of pancreatic beta cells could lead to the decline in function or even the destruction of pancreatic beta cells, leading to Diabetes Mellitus and long-term metabolic alterations (17).

In our cohort, over 35% of Non-DM and over 65% of DM patients presented above the reference range for fasting blood glucose. Corroborating with a previous investigation that identified a need for increasing blood glucose drugs after COVID-19 in DM patients (18). Although Non-DM patients can develop hyperglycemia during infections (5, 19), it was expected to return to normal levels after the hospital discharge.

To confirm a prolonged blood glucose elevation, we performed a glycated hemoglobin test and identified over 50% of the Non-DM with above the reference levels. Which could increase the susceptibility to other infections post-COVID-19 (7).

Importantly, we did not identify a correlation between the COVID-19 severity and the long-term alterations on blood glucose, since only five Non-DM patients were hospitalized in the intensive care unit with mechanical ventilation.

C-peptide is a useful and widely used method of assessing pancreatic beta cell function. Since in our cohort both groups presented normal or above the reference range for c-peptide, it is expected that pancreatic beta cell functions are preserved or hyper-activated, possibly indicating a metabolic dysfunction and insulin resistance (20).

Chronic kidney disease could impact the c-peptide measurement, due to the c-peptide is metabolized by the kidneys (21), but in our cohort, Non-DM did not present alterations on kidney biomarkers or possess diagnosed kidney disfunction.

## Conclusion

Our results demonstrated that COVID-19 generates a hyperinflammatory and hypercoagulation syndrome with similar severity in DM and Non-DM patients. 180 days post-hospitalization all patients presented normal levels in all laboratory data, indicating that SARS-CoV-2 infection did not generate chronic low-level inflammation. Several DM and Non-DM patients presented elevated levels of fasting blood glucose and glycated hemoglobin 180 days post-COVID-19, and this elevation was not correlated with impaired beta cells function. Our investigation has several limitations such as: patients infected with SARS-CoV-2 variants may present a different progression and long-term effects; not all patients from our cohort returned for posterior evaluation; further mechanism investigations must be performed to better understand how COVID-19 may affect glucose metabolism in DM and non-DM patients. In summary, our results put into perspective the necessity for all patients post-COVID-19 to perform long-term blood glucose tests to assess the risk of developing type 2 DM and glycemic control.

## Data availability statement

The raw data supporting the conclusions of this article will be made available by the authors, without undue reservation.

## Ethics statement

The studies involving human participants were reviewed and approved by Ethics Committee of HCFMUSP (no.30800520.7.0000.0068-2020). The Ethics Committee waived the requirement of written informed consent for participation.

## Author contributions

All authors contributed to the conception, writing, and review of the article and approved the submitted version.

## Funding

This work was supported by RA holds a fellowship from Fundação de Amparo à Pesquisa do Estado de São Paulo (FAPESP) No. 19/02679-7, MS holds a grant from Fundação de Amparo à Pesquisa do Estado de São Paulo (FAPESP) No. 20/13148-0, 19/25119-7, and AD holds a grant from Coordenação de Aperfeiçoamento de Pessoal de Nível Superior (CAPES) No. 88887.503842/2020-00.

## Conflict of interest

The authors declare that the research was conducted in the absence of any commercial or financial relationships that could be construed as a potential conflict of interest.

## Publisher's note

All claims expressed in this article are solely those of the authors and do not necessarily represent those of their affiliated organizations, or those of the publisher, the editors and the reviewers. Any product that may be evaluated in this article, or claim that may be made by its manufacturer, is not guaranteed or endorsed by the publisher.
